# Study of the Relationship between Haze Performance and Fractal Dimension in Micro-Sized Segregated Liquid Crystals Embedded in a Polymer Matrix Consisting of a Thiol-ene Prepolymer and a Multi-Functional Acrylate

**DOI:** 10.3390/polym13244421

**Published:** 2021-12-16

**Authors:** Ju-Yong Kim, Suk-Won Choi

**Affiliations:** Department of Advanced Materials Engineering for Information & Electronics, and Integrated Education, Institute for Frontier Science & Technology (BK21 Four), Kyung Hee University, Yongin 17104, Gyeonggi-do, Korea; kjuyong0818@naver.com

**Keywords:** optical haze, liquid crystal, thiol-ene polymers, multi-functional acrylates, fractal

## Abstract

Micro-sized segregated liquid crystals (MSLCs) surrounded by a polymer medium can be used for haze film applications. When incident light passes through the MSLC film, the microsized particles act as light scattering centers. In this study, the results of the addition of a multi-functional acrylate to a commercial thiol-ene prepolymer system, as well as the morphology of (LC) droplets, fractal dimension (*D*), and the optical haze performance of the micro-sized segregated LCs formed by UV-initiated photopolymerization, are reported. With increasing fraction of the multi-functional acrylate within the host polymer matrix, the small scattering centers (LC droplets) also increase, giving rise to a large optical haze in the prepared film. The optical haze can be characterized by the *D* of the associated LC droplet morphology in the films. The optical haze and *D* exhibit a strong correlation; thus, a qualitative prediction of the optical haze is possible via geometric fractal analysis.

The self-aggregating and self-assembling properties of liquid crystal (LC) materials open the possibility for the emergence of novel materials [1,2]. Polymer-dispersed LCs (PDLCs), consisting of LC droplets dispersed in a polymer matrix can be electrically switched from an opaque (light-scattering) state to a transparent (non-scattering) state for smart windows or display applications [3]. Recently, we reported micro-sized segregated LCs (MSLCs) surrounded by a polymer medium [2]. When incident light passes through the MSLC film, the micro-sized LC droplets, which are phase-segregated from the host polymer matrix act as light scattering centers. This characteristic originates from the refractive index mismatch between the LCs and the polymer. The MSLCs are similar to PDLCs in the sense that phase-segregated LC droplets are dispersed in a polymer matrix. However, MSLCs are only designed for optical haze films using the scattering features of the PDLCs without applying electric fields [2]. Hence, our fabricated films are referred to as MSLCs instead of PDLCs to highlight the functional differences between the two films.

Thiol-ene polymers are prepared by a combination of step-growth and free-radical polymerizations between aliphatic thiols and allyl (or vinyl) monomers containing carbon-carbon double (C=C) bonds [4]. Thiol-ene polymers find use in various applications, such as surface coatings and adhesives. This is because the thiol-ene polymerization scheme is useful for obtaining synthesized polymers with a high conversion rate and uniform cross-link densities under ambient-pressure, room-temperature, and solvent-free conditions [5]. Herein, we report the results of a study conducted on the addition of a multi-functional acrylate to a commercial thiol-ene prepolymer system, and the morphology of LC droplets, fractal dimension (*D*), and the optical haze performance of MSLCs formed by UV-initiated photopolymerization. Thus, we fabricated MSLC films by adding a multi-functional acrylate to thiol-ene prepolymers as a host polymer matrix for the LC phase segregation. We evaluated the optical haze performance of the MSLCs as a function of the doping fraction of the multi-functional acrylate prepolymer. As the fraction of the multi-functional acrylate within the host polymer matrix increases, the LC droplet size decreases while the LC droplet number increases, resulting in an enhanced optical haze performance of the MSLC film. In addition, we demonstrated that the optical haze performance can be characterized by the *D* of the LC droplet morphology in the MSLC films. Interestingly, the optical haze performance and *D* in the MSLC films exhibit a strong correlation. This strong correlation was also confirmed in the MSLC films with different film thicknesses.

First, we prepared four host formulations with different ratios of multi-functional acrylate blended with a thiol-ene prepolymer. Commercially available NOA 88 (Norland Inc., Cranbury, NJ, USA) and trimethylolpropane triacrylate (TMPTA, Sigma-Aldrich Seoul, Korea) were used as the thiol-ene prepolymer and multifunctional acrylate, respectively. The chemical structure of TMPTA is shown in Figure 1a. The commercially available low-molecular nematic LC mixture (HTW109100-100, HCCH, Nanjing, China) was used as guest. This LC mixture exhibited high stability against processing conditions, such as UV irradiation and curing temperature, during the fabrication of MSLC films. The mixing ratio of the host (thiol-ene prepolymer and multi-functional acrylate) and guest (nematic LC) was fixed at 70:30 wt.% without solvent. Detailed mixing ratios for the four mixtures (MSLC-0, MSLC-6, MSLC-12, and MSLC-20) are summarized in Table 1. Since commercial NOA 88 contains a proprietary photo-initiator, there was no need for an extra initiator. The prepared mixtures were inserted by capillary action into cells consisting of two glass substrates without surface treatment. The cell gaps between the two substrates were maintained using 30 μm glass beads. The cells filled with the homogeneous mixture were exposed to a UV light (365 nm, 6 W) for 15 min at 55 °C under ambient pressure. Once the polymerization reaction of the blended prepolymer was initiated in the homogeneous mixture, spontaneous polymerization-induced phase separation occurred between the host and guest. As a result, the guest came out of the homogenous phase and began to form LC droplets. The LC droplets grew until the UV-initiated polymerizable prepolymer became sufficiently solid to trap the LCs and prevent them from moving easily [6]. The extraordinary refractive index of the guest used here was *n_e_* = 1.706 (at 20 °C, 589 nm), and the refractive index of the host was *n* = 1.56. This refractive index mismatch between the LCs and the polymer resulted in light scattering [7]. There is a high probability that the size of the droplet becomes smaller than the visible wavelength scale at low LC concentration, similar to the present case. However, the evaluated size of the droplet was of the order of several microns, which is relatively large. This relatively large size can be attributed to the polymerization conditions, especially the UV curing temperature. It is empirically known that the average droplet size increases when prepolymer is polymerized at low curing temperature. Thus, with a decrease in the curing temperature, the degree of matrix formation decreases, enhancing phase separation which yields bigger droplet sizes [8]. It should be noted that the present system was polymerized at 55 °C, which is a relatively low curing temperature.

Figure 1b presents histograms of the LC droplet size distribution in the MSLC-0, MSLC-6, MSLC-12, and MSLC-20 films, respectively. Typical polarized optical microscopy (POM) images of the four films are also provided in the insets to Figure 1b. The LC droplet size distributions were obtained from the POM images processed by the software ImageJ, which was developed at the National Institutes of Health (Bethesda, MD, USA) and is a freely available image processing and analysis program [9,10]. As shown in Figure 1b, the mean LC droplet size decreases, and the LC droplet number increases on increasing the fraction (up to 20 wt.%) of the multi-functional acrylate within the host polymer matrix. This is because the crosslink density within the host polymer matrix increases on adding multi-functional acrylates into the thiol-ene prepolymers. Acrylate incorporation in the thiol-ene matrix increases the rubbery modulus of the system owing to the heterogeneous distribution of cross-linked regions; the modulus is proportional to the number density of network strands between the crosslinks [11,12]. The polymer network with high crosslink density exhibits a low ability to swell with LCs [13]; thus, LCs occupying these polymer regions are large in number, but small in size.

The *D* is an effective parameter for analyzing complex structures in several areas of science [14]. Fractal dimensional analysis of the LC droplets in the MSLC films was performed using POM images, as shown in Figure 2. The self-similar nature refers to the fact that if a portion of a system is magnified, the overall structure would resemble the original piece irrespective of the magnification or size of the original portion [14]; this self-similarity was quantified using the *D*. Grayscale versions of the original color POM images were converted to binary data, and fractal image analysis was also performed with the plugin FracLac for ImageJ [10] using the box dimension method for each POM image of the four MSLC films. This method yields the **D** from the exponent in the following proportionality [14]:

(1) $$N\left(d\right)\propto \frac{1}{d^{D}}$$

where *N*(*d*) is the number of boxes of length d occupied by the dataset (here, the LC droplets). The *D* varied between 1 and 2. *D* = 1 indicates a single LC droplet and *D* = 2 corresponds to a complete space-filling structure (i.e., LC droplets filling all of the investigated sandwich cells) [14].

Figure 3a presents the evaluated *D* of the LC droplet surrounded by polymer networks as a function of the fraction of the multi-functional acrylate within the host polymer matrix. As shown in Figure 3a, as the fraction of the multi-functional acrylate increased, the *D* also increased. In the case of acrylate incorporation in the thiol-ene prepolymer, the thiol-ene polymerization process as well as another polymerization occurred simultaneously via the blended multi-functional acrylate [15]. This gave rise to the formation of a polymerized network via an acrylate-acrylate polymerization along with the thiol-ene polymerized network [15]. It yielded long, complex, and chained polymerized structures as a thiol-ene-acrylate network [16]. The fractal structures of polymer networks are expected because of the presence of thiol and acrylate in these systems [5,17,18]. Hence, the fractal structures of the polymer networks yield that of the LC droplets because the LC droplets are embedded in the polymer networks consisting of thiol-ene-acrylate networks. The optical haze profiles of the four MSLC films prepared are shown in Figure 3b. Optical haze can be used to manipulate light behavior and is given by:

Optical haze = *DT/TT × 100* (2)

where *DT* is the diffusely transmitted light, and *TT* is the total transmitted light [7]. The *TT* and *DT* spectra of the MSLC films were measured using a haze meter (HAM-300, Everfine, Hangzhou, China) with an integrating sphere (inner diameter: 60 mm). The optical haze was calculated using Equation (2) Figure 3c shows the plot for average optical haze over the wavelength range of 450 ≤ λ ≤ 800 nm as a function of the blended amount of multi-functional acrylate (TMPTA) within the host polymer matrix. As expected, the optical haze performance was enhanced because small scattering centers (LC droplets) increased on increasing the fraction of the multi-functional acrylate. Figure 3d shows the plot for the average optical haze as a function of the *D* of the LC droplets in the four MSLC films. Interestingly, the average optical haze strongly correlated with the *D* of the LC droplets in the MSLC films.

To validate the strong correlation between the *D* and the optical haze, we prepared four MSLC-20 films with different film thicknesses: 5, 10, 20, and 30 μm. MSLC-20 consisted of 20 wt.% of multi-functional acrylate TMPTA, 50 wt.% of thiol-ene prepolymer NOA 88, and 30 wt.% of LC, as listed in Table 1. Figure 4a shows the *D* of the LC droplet surrounded by polymer networks according to the thickness of the MSLC-20 films. Using a method similar to that explained in Figure 2, the fractal dimensional analysis of the four MSLC films with different film thicknesses was carried out. Figure 4b shows the optical haze profiles of the four MSLC films with different film thicknesses. Figure 4c shows the plot for the average optical haze over the wavelength range of 450 ≤ λ ≤ 800 nm as a function of MSLC-20 film thickness. As shown in Figure 4a,c, the *D* and the optical haze performance increased on increasing the film thickness. This is also due to the increase in scattering centers (LC droplets) on increasing the film thickness. The average optical haze as a function of the *D* of the LC droplets in the four MSLC films prepared is also plotted in Figure 4d. Even in this case, the average optical haze was strongly correlated with the *D* of the LC droplets in the MSLC films, as shown in Figure 3d. It is elucidated that we can qualitatively predict the optical haze performance via the geometric fractal analysis in the MSLCs

In conclusion, we fabricated the MSLC films by adding a multi-functional acrylate to thiol-ene prepolymers as a host polymer matrix for the LC phase segregation. We evaluated the optical haze performance of the MSLCs as a function of the doping fraction of the multi-functional acrylate prepolymer. On increasing the fraction of the multi-functional acrylate within the host polymer matrix, small scattering centers (LC droplets) increased, resulting in an enhanced optical haze of the MSLC film. We also demonstrated that the optical haze performance can be characterized by the *D* of the LC droplet morphology in the MSLC films. Interestingly, the optical haze performance and *D* exhibited a strong correlation in the MSLC films with different fractions of multi-functional acrylate. This strong correlation was also confirmed in the MSLC films with different film thicknesses. Our approach indicates that it is possible to qualitatively predict the optical performance via geometric fractal analysis in complex polymeric composites, such as MSLC films.

## Figures and Tables

**Figure 1:**
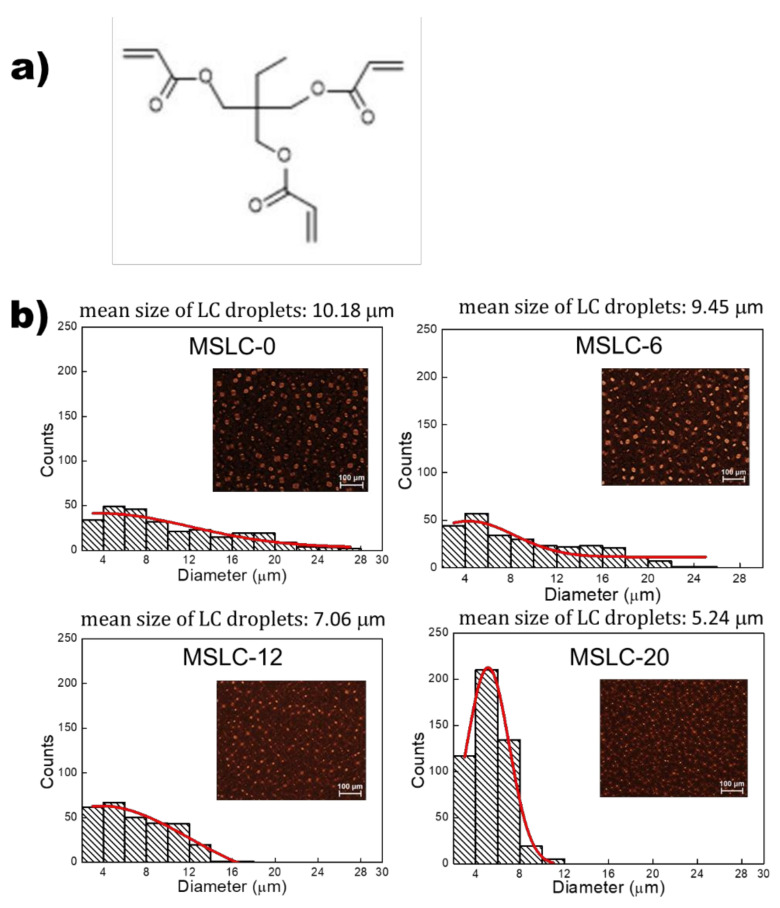
(**a**) Chemical structure of the multi-functional acrylate TMPTA. (**b**) Histograms of the LC droplet size distribution in the MSLC-0, MSLC-6, MSLC-12, and MSLC-20 films, respectively. Typical POM images of the four films are also provided in the insets.

**Figure 2:**
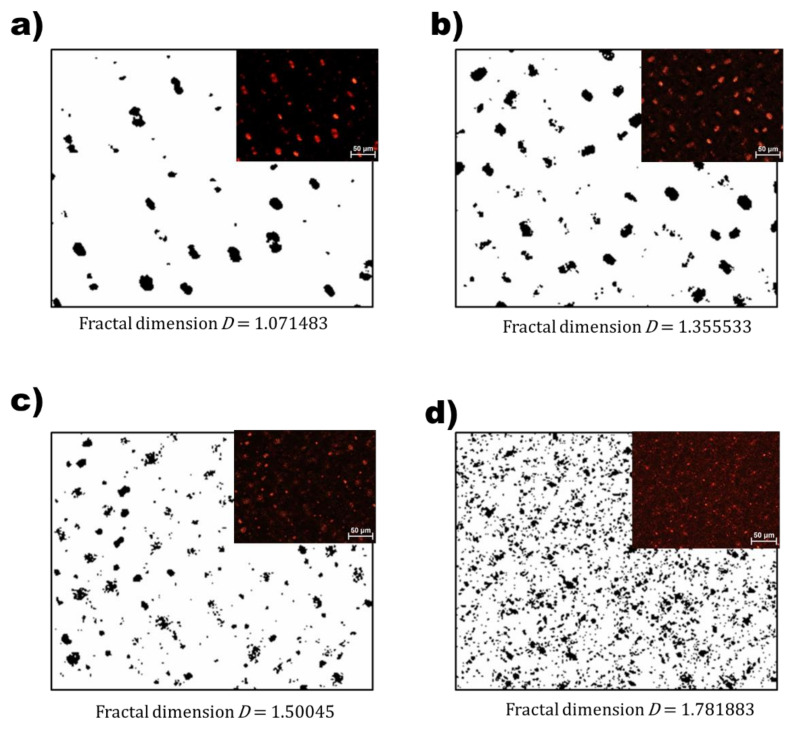
Grayscale images used for geometric fractal analysis; (**a**) MSLC-0, (**b**) MSLC-6, (**c**) MSLC-12, and (**d**) MSLC-20. Original color POM images of the four films are also provided in the insets.

**Figure 3:**
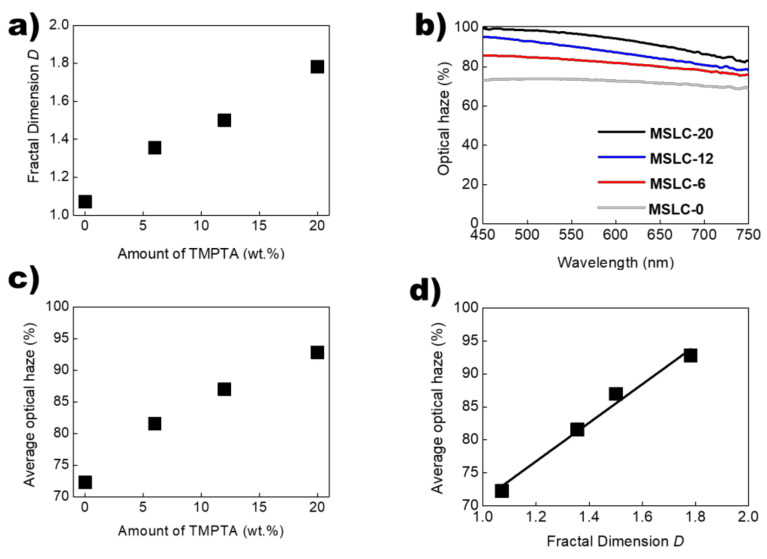
(**a**) *D* of the LC droplet surrounded by polymer networks as a function of the fraction of the multi-functional acrylate within the host polymer matrix. (**b**) Optical haze profiles of the four MSLC films prepared. (**c**) Average optical haze over the wavelength range of 450 ≤ *λ* ≤ 800 nm as a function of the blended amount of multi-functional acrylate TMPTA within the host polymer matrix. (**d**) Plot of average optical haze versus the *D* of the LC droplets in the four MSLC films.

**Figure 4:**
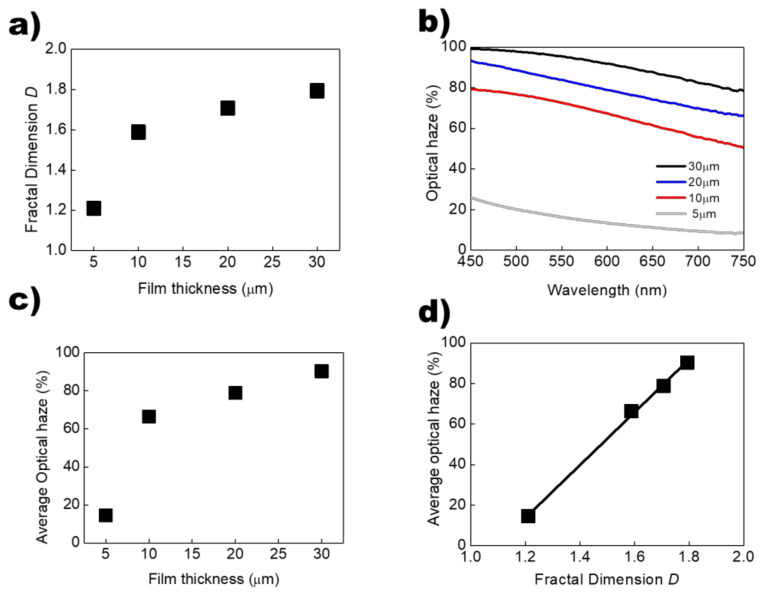
(**a**) *D* of the LC droplet surrounded by polymer networks as a function of film thickness. (**b**) Optical haze profiles of the four MSLC films prepared with different thicknesses. (**c**) Average optical haze over the wavelength range of 450 ≤ *λ* ≤ 800 nm as a function of film thickness. (**d**) Plot of average optical haze versus the *D* of the LC droplets in the prepared MSLC films with different thicknesses.

**Table 1:** Mixing ratios (wt.%) of the four mixtures prepared in this study.

	NOA 88	TMPTA	HTW 109100-100
MSLC-0	70	0	30
MSLC-6	64	6	30
MSLC-12	58	12	30
MSLC-20	50	20	30

## Data Availability

The data presented in this study are available on request from the corresponding author.
